# Diabetes in South African older adults: prevalence and impact on quality of life and functional disability – as assessed using SAGE Wave 1 data

**DOI:** 10.1080/16549716.2018.1449924

**Published:** 2018-04-27

**Authors:** Mahmoud Werfalli, Reshma Kassanjee, Sebastiana Kalula, Paul Kowal, Nancy Phaswana-Mafuya, Naomi S. Levitt

**Affiliations:** a Chronic Disease Initiative in Africa, Division of Diabetic Medicine and Endocrinology Department of Medicine, University of Cape Town, Cape Town, South Africa; b Department of Statistical Sciences, University of Cape Town, Cape Town, South Africa; c Albertina & Walter Sisulu Institute of Ageing in Africa, Division of Geriatric Medicine, Department of Medicine, University of Cape Town, Cape Town, South Africa; d WHO Study on global AGEing and adult health (SAGE), Geneva, Switzerland; e Research Centre for Generational Health and Ageing, University of Newcastle, Newcastle, NSW, Australia; f Office of the Deputy Vice Chancellor, Research and Innovation, North West University, Potchefstroom, South Africa; g HIV/AIDS/STI/TB Research Programme, Human Sciences Research Council, Pretoria, South Africa

**Keywords:** Global ageing, diabetes, prevalence, disability, health-related quality of life

## Abstract

**Background**: Diabetes is a chronic disease with severe late complications. It is known to impact the quality of life and cause disability, which may affect an individual’s capacity to manage and maintain longer-term health and well-being.

**Objectives**: To examine the prevalence of self-report diabetes, and association between diabetes and each of health-related quality of life and disability amongst South Africa’s older adults. To study both the direct relationship between diabetes and these two measures, as well as moderation effects, i.e. whether associations between other factors and these measures of well-being differed between individuals with diabetes and those without.

**Methods**: Secondary analyses of data on participants aged 50 years and older from the Study on global AGEing and adult health (SAGE) in South Africa Wave 1 (2007–2008) were conducted. Prevalence of self-reported diabetes was assessed. Multivariable regressions describe the relationships between each of quality of life (WHOQoL) and disability (WHODAS), and diabetes, while controlling for selected socio-demographic characteristics, health risk behaviours and co-morbid conditions. In the regression models, we also investigated whether diabetes moderates the relationships between these additional factors and WHOQoL/WHODAS.

**Results**: Self-reported diabetes prevalence was 9.2% (95% CI: 7.8,10.9) and increased with age. Having diabetes was associated with poorer WHOQoL scores (additive effect: −4.2; 95% CI: −9.2,0.9; p-value <0.001) and greater disability (multiplicative effect: 2.1; 95% CI: 1.5,2.9; p-value <0.001). Lower quality of life and greater disability were both related to not being in a relationship, lower education, less wealth, lower physical activity and a larger number of chronic conditions.

**Conclusions**: Diabetes is associated with lower quality of life and greater disability amongst older South Africans. Attention needs to be given to enhancing the capacity of health systems to meet the changing needs of ageing populations with diabetes in SA as well as facilitating social support networks in communities.

## Background

Populations are ageing worldwide, and the pace of the demographic transition is fastest in developing countries, including those in Africa [1]. The number of people aged 60 years and older in sub-Saharan Africa is projected to be double that in Northern Europe by 2050, and to rise faster than any other region, increasing almost fourfold: from 46 million in 2015 to 157 million by 2050 [2]. South Africa (SA) has one of the largest ageing populations in Africa with more than 1 in 6 individuals aged 50 years and older. Almost 8% of the current population is aged 60 years and older equating to approximately 4.2 million people and is projected to rise. The number of those aged 60 years and older is projected to rise to 10.1 million (15%) by 2050 [3].

Concomitant with people living longer and an expanding older section of the population is an increase in chronic morbidity. Type 2 diabetes mellitus is typically more common in older than younger adults. Rapid urbanisation is contributing to higher disease prevalence in numerous African countries with prevalence higher in older adults than in any other age groups [4]. Untreated or inadequately managed diabetes may lead to long-term complications, including blindness, kidney disease, peripheral neuropathy and macrovascular disease, in turn leading to amputations, stroke and heart attacks. Such complications contribute significantly to mortality, morbidity and health system costs [5]. Diabetes is currently the seventh leading cause of morbidity and mortality in South Africa, due to trends in obesity, poor diet, high fasting blood glucose and low physical activity levels [6]. Health care systems in many African countries are hard-pressed to provide adequate care to older patients with diabetes or indeed to prevent or delay the onset of the disease, in old age or earlier in the life course. Complications that arise from diabetes, particularly through inadequate management, have the potential to impact the quality of life [6].

The World Health Organization (WHO) defined the health-related quality of life (HRQoL) as ‘the individual’s perception of their position in life in the context of the cultural and value systems in which they lived and related to their goals, expectations, standards and concerns [7]’. Provision of quality health care as a means to maintain the quality of life and well-being remains a challenge in South Africa, both in urban areas and in rural areas. The current public health care system is geared towards management of single acute diseases and not clients with complex multiple chronic conditions, such as more often seen in older than in younger patients [8]. Further complicating the situation, older clients’ dissatisfaction with service delivery at public healthcare facilities has been widely documented and pertains mainly to access barriers to care [8]. Adequate management of diabetes to prevent complications that impact the quality of life requires a supportive, accessible health care system, especially at primary care level [7,8].

Quality of life (QoL) and disability are powerful predictors of a person’s capacity to manage and maintain longer-term health and well-being. QoL is a form of evaluative well-being that refers to perceptions of the quality or goodness of one’s life or one’s overall life satisfaction [9]. Psychological well-being and health are closely related, possibly becoming more important at older ages [9]. Age-related disability is an umbrella term that encompasses decrements in bodily function, task performance and involvement in life situations [10]. Both HRQoL and disability are health-related outcomes which reflect the overall health of an individual, including those with diabetes [10]. The HRQoL reported by older people with chronic diseases such as diabetes may reflect its restrictive management regimen, disease sequelae, and impact of associated co-morbidities [11].

This study aimed to fill a gap in knowledge regarding the relationship between diabetes and the well-being and functioning of South Africans aged 50 years and older. Using data from Wave 1 of the WHO Study on global AGEing and adult health (SAGE), we investigated the prevalence of self-report diabetes and the association between diabetes and each of the WHO Quality of Life (WHOQoL) and WHO Disability Assessment Schedule (WHODAS) scores. In the analysis, we controlled for selected socio-demographic characteristics, health risk behaviours and the presence of co-morbid conditions. We also explored whether the associations between these additional factors and WHOQoL and WHODAS differed between individuals with diabetes and those without – i.e. investigated the moderation of effects by diabetes status.

## Methods

### Data source

Secondary analyses were undertaken of data from the WHO Study on global AGEing and adult health (SAGE), a longitudinal, multi-country study conducted in China, Ghana, India, Mexico, the Russian Federation and South Africa. SAGE collected individual-level data from nationally representative household samples of older adults (50 years and older) using a multistage cluster sampling design and included a smaller sample of younger adults (aged 18–49 years) for comparison purposes [12]. SAGE Wave 2 was conducted in 2014/2015 but data for this study was not available to the public at the time the current study was conducted [13].

Cross-sectional data from Wave 1 (2007/08) in South African adults aged 50 years and older were analysed. The sample comprised 4037 participants, 3836 (90.9%) of whom were 50 years and older. The household response rate for South Africa was 67%, and individual response rates were 77% [12]. All participants aged 50 years and older in the selected older households were invited to complete a personal interview. Proxy respondents (through cognitive screening) were identified for a subsample unable to complete an interview. Face-to-face interviews using a standardised questionnaire were used to collect information on socio-demographic characteristics, disability, subjective well-being, and other health measures and behavioural risk factors. Post-stratified weights were used to ensure that estimates were nationally representative [14].

### Measurements

The following measures were used to assess quality of life and level of disability:

- Subjective well-being, or quality of life, was measured using the eight-item WHO Quality of Life Instrument (WHOQoL) [14]. Outcomes from the eight questions were summed to obtain an overall WHOQoL score which was transformed to a 0–100 scale, where a score of 0 represents the worst quality of life, and a maximum score of 100 represents the highest quality of life.
- Disability level was measured using the 12-item WHO Disability Assessment Schedule (WHODAS) encompassing 6 domains of functioning; questions were summed to get an overall WHODAS score, initially a count ranging from 0 to 36. The score can be transformed onto a 0–100 scale, with 0 as no disability and 100 maximum disability [15].

The relationship between diabetes and WHOQoL and WHODAS was investigated, where diabetes status was determined through self-report, using the question ‘Has a health professional/doctor ever told you that you have diabetes?’ Additionally, participants reporting taking medication for diabetes were considered as having diabetes (or high blood sugar).

The additional variables considered were as follows:

- Selected socio-demographic characteristics were used as covariates in the analyses. These include age groups (50–59 years, 60–69 years, 70 years and older), sex (male, female), marital status (single, married/cohabiting, separated/divorced, widowed) and years of education (0–5 years, 6–12 years, ≥ 13 years). ‘Same location’ was determined using the question ‘Have you always lived in this village/town/city?’ Employment status (ever worked, never worked) and wealth quintiles (lowest to highest) were also considered. Since direct socio-economic measures are available, ethnicity, historically used as a proxy for this in SA, is excluded from the models presented below.
- Health-related variables included the use of tobacco (ever smoked, never smoked) and the use of alcohol (never drank, ever drank). A categorical variable indicating low, medium and high physical activity levels was constructed from the Global Physical Activity Questionnaire (GPAQ) included within SAGE [16].
- Self-reported chronic conditions were assigned based on similar questions to those used for diabetes but applied to the following conditions: arthritis, stroke, angina, chronic lung disease, asthma, hypertension, cataracts and depression. A count variable was also generated for each participant (collapsed into zero, one, two or more chronic conditions).

### Ethical clearance

SAGE Wave 1 was approved by the HSRC Research Ethics Committee (South Africa) and the National Department of Health. SAGE was also approved by the WHO Ethical Review Committee. Respondents provided written informed consent. WHO SAGE granted the investigators’ permission for access to the de-identified data for secondary analysis in June 2014.

### Statistical analyses

Data were analysed using R (version 3.1.3) [17], and the sampling design and probability weights were taken into account using R’s ‘survey’ package [18]. When additional model options were required, Stata (Stata/MP 13.1) [19] was used, utilising the suite of ‘svy’ commands. The methodology is briefly summarised below, and some further details provided in Appendix E of the supplementary material.

WHOQoL and WHODAS were analysed in turn, using generalised linear models. For WHOQoL, a standard multiple linear regression model was implemented, while for WHODAS, a zero-inflated negative binomial regression model with a log link was fitted to the data. Model choice was guided by exploration of the data and comparing a few candidate models, and confirmed to be adequate through residual diagnostic plots and comparisons of observed and model-fitted scores (see Appendices C, D and H for some plots of the data and comparisons).

The regression models aim to estimate the relationship between diabetes and each of the two measures of well-being, namely WHOQoL or WHODAS, while controlling for the socio-demographic, health risk behaviour and chronic conditions variables listed above. They also aim to understand whether the relationships between these factors and the well-being measures are modified by the presence of diabetes.

Two sets of p-values are reported in results for each factor: (a) The p-value for ‘factor’ relates to testing whether that factor is related to well-being in any way. (b) The p-value for the ‘moderation effect’ relates to testing whether diabetes moderates the relationship between that factor and the well-being score – i.e. whether the relationship is different between individuals with diabetes and those without. Because testing for moderation is of interest, the dataset was not stratified into those with diabetes and those without, but rather all data used in the model.

In the initial fitting of the models, diabetes was allowed to moderate all effects and therefore distinct ‘diabetic’ and ‘non-diabetic’ group effect sizes are reported in the tables. To reduce the size of the model, for a given factor, when the p-value for the moderation effect was greater than 0.1, the moderation term was removed, and in these cases just one ‘all subjects’ effect size is reported below.

To account for the zero-inflation for WHODAS (i.e. the excess of zero values compared to what is expected using a negative binomial distribution) an extra set of parameters was estimated and is reported. These odds ratios describe the impact of the factor on the extent of these zero values (no moderation by diabetes status allowed). Again, when the terms did not seem necessary (using a threshold of 0.1 on p-values) they were removed from the model, and therefore not all factors have associated odds ratios.

List-wise deletion of observations with missing values resulted in 2848 observations for WHOQOL and 2866 observations for WHODAS. The most common missing values were: 570 for education, 492 for place of residence and 123 for physical activity.

## Results

The overall prevalence of self-reported diabetes was 9.2% (95% CI: 7.8,10.9). It increased with age: from 7.1% (95% CI: 5.4,9.2) amongst 50–59-year olds, to 10.6% (95% CI: 8.0,14.0) amongst 60–69-year olds, to 12.4% (95% Cl: 9.1,16.7) amongst those 70 years and older.

The socio-demographic characteristics, health risk behaviours and chronic conditions of all the participants, also stratified by diabetes status, are described in Table 1. Half of the respondents were aged between 50–59 years (mean age 61.6 years, SD 9.5), 56% were female, 56% were in a current partnership, and 46% had less than 5 years of formal education. The majority of respondents in both groups engaged in only low levels of physical activity (60%). People in the diabetes group had at least twofold higher rates of coexisting self-reported chronic conditions (arthritis, stroke, angina, chronic lung disease, asthma, hypertension, cataracts and depression) compared to the non-diabetes group.

**Table 1.** Number and percentage of participants by socio-demographic characteristics, self-reported health behaviours and co-morbidities.

		n (%)		
Factor^a^	Category	All subjects^b^	Ever diagnosed with with diabetes	No self-reported diabetes
All participants		3836 (100.0)	341 (100.0)	3495 (100.0)
Diabetes	No	3362 (90.8)		3362 (100.0)
	Yes	341 (9.2)	341 (100.0)	
Sex	Female	2146 (55.9)	228 (67.0)	1850 (55.0)
	Male	1690 (44.1)	113 (33.0)	1512 (45.0)
Age	50–59 years	1913 (49.9)	130 (38.1)	1711 (50.9)
	60–69 years	1174 (30.6)	121 (35.6)	1021 (30.4)
	70+ years	749 (19.5)	89 (26.2)	630 (18.7)
Marital status	Single	539 (14.3)	45 (13.3)	471 (14.3)
	Married/cohabiting	2108 (55.9)	170 (50.2)	1872 (56.7)
	Separated/divorced	223 (5.9)	9 (2.8)	206 (6.2)
	Widowed	900 (23.9)	114 (33.8)	754 (22.8)
Years of education	0–5 years	1418 (46.4)	95 (35.0)	1288 (47.6)
	6–12 years	1369 (44.8)	161 (58.9)	1168 (43.2)
	13+ years	269 (8.8)	17 (6.1)	249 (9.2)
Same location	Yes	2141 (68.1)	190 (66.9)	1889 (67.9)
	No	1002 (31.9)	94 (33.1)	892 (32.1)
Ever worked	Yes	3237 (85.4)	286 (84.2)	2878 (85.6)
	No	553 (14.6)	54 (15.8)	484 (14.4)
Wealth quintile	Poorest	790 (20.7)	39 (11.6)	704 (21.0)
	Second	759 (19.9)	50 (14.8)	684 (20.5)
	Middle	696 (18.2)	60 (17.6)	627 (18.7)
	Fourth	757 (19.8)	87 (25.5)	650 (19.4)
	Richest	815 (21.3)	103 (30.4)	681 (20.3)
Tobacco	No	2459 (66.4)	268 (78.8)	2188 (65.2)
	Yes	1242 (33.6)	72 (21.2)	1169 (34.8)
Alcohol	No	2765 (74.7)	294 (86.5)	2468 (73.5)
	Yes	934 (25.3)	46 (13.5)	889 (26.5)
Physical activity	Low	2154 (60.1)	220 (68.0)	1932 (59.3)
	Moderate	436 (12.2)	41 (12.8)	395 (12.1)
	High	996 (27.8)	62 (19.2)	933 (28.6)
Arthritis	No	2788 (75.3)	188 (55.1)	2600 (77.3)
	Yes	915 (24.7)	153 (44.9)	762 (22.7)
Stroke	No	3553 (96.0)	317 (93.0)	3235 (96.3)
	Yes	149 (4.0)	24 (7.0)	125 (3.7)
Angina	No	3508 (94.8)	299 (87.6)	3209 (95.5)
	Yes	194 (5.2)	42 (12.4)	152 (4.5)
Lung disease	No	3596 (97.1)	312 (91.7)	3283 (97.7)
	Yes	106 (2.9)	28 (8.3)	78 (2.3)
Asthma	No	3523 (95.1)	308 (90.5)	3213 (95.6)
	Yes	181 (4.9)	32 (9.5)	148 (4.4)
Depression	No	3596 (97.1)	322 (94.6)	3272 (97.4)
	Yes	106 (2.9)	18 (5.4)	88 (2.6)
Hypertension	No	2580 (69.7)	105 (30.7)	2474 (73.6)
	Yes	1124 (30.3)	236 (69.3)	888 (26.4)
Cataracts	No	3528 (95.6)	290 (86.8)	3237 (96.5)
	Yes	163 (4.4)	44 (13.2)	118 (3.5)
# chronic conditions	0	1846 (50.1)	52 (15.4)	1793 (53.5)
	1	1122 (30.5)	114 (34.0)	1008 (30.1)
	2	460 (12.5)	105 (31.4)	355 (10.6)
	3+	256 (7.0)	64 (19.2)	192 (5.7)

^a^For a factor, sum of counts for categories do not necessarily equal ‘All subjects’ counts because of missing data.

^b^Sum of diabetes and non-diabetes counts do not necessarily equal ‘All subjects’ counts because of missing data.

To further describe the data, see Appendix A and Appendix B of the supplementary material for summary statistics for WHOQoL and WHODAS, stratified by diabetes status and other covariates.

The associations between factors and the average WHOQoL scores, as modelled using a multivariable regression, are shown in Table 2. The additive effect or regression coefficient describes the change in the average WHOQoL score associated with a change in factor (out of the reference category into another category) – and therefore positive values indicate increases in quality of life.

**Table 2.** Association of diabetes, socio-demographic characteristics, self-reported health behaviours and co-morbidities with WHOQOL (0–100).

		Additive effect/regression coefficient (95% CI)			P-values^a^	
Factor	Category	All subjects	Non-diabetic group	Diabetic group	Factor	Moderation effect^b^
Diabetes	No (ref)				<0.001	N/A
	Yes	−4.2 (−9.2;0.9)				
Sex	Female (ref)				0.047	
	Male	−1.4 (−2.8;-0.0)				
Age	50–59 years (ref)				0.046	
	60–69 years	1.7 (0.3;3.1)				
	70+ years	1.4 (−0.3;3.1)				
Marital status	Single	−2.6 (−4.8;-0.5)			0.016	
	Married/cohabiting (ref)					
	Separated/divorced	−1.2 (−4.2;1.8)				
	Widowed	−2.5 (−4.1;-0.8)				
Years of education	0–5 years (ref)				0.003	
	6–12 years	2.2 (0.6;3.7)				
	13+ years	5.0 (1.9;8.0)				
Same location	Yes (ref)				0.703	
	No	0.3 (−1.2;1.8)				
Past work	Yes (ref)				0.003	0.137
	No		−4.2 (−6.6; −1.8)	−1.1 (−4.6;2.3)		
Wealth quintile	Lowest		−6.8 (−9.1; −4.4)	−9.4 (−14.4; −4.3)	<0.001	0.139
	Second		−2.7 (−5.2; −0.3)	−5.9 (−12.3;0.6)		
	Middle (ref)					
	Fourth		0.5 (−1.5;2.5)	−2.6 (−6.6;1.4)		
	Highest		6.9 (4.2;9.6)	1.3 (−2.3;4.8)		
Tobacco	No (ref)				0.62	
	Yes	0.4 (−1.2;2.1)				
Alcohol	No (ref)				0.061	
	Yes	−1.8 (−3.6;0.1)				
Physical activity	Low		−3.3 (−5.3;-1.3)	0.2 (−3.0;3.4)	<0.001	0.028
	Moderate (ref)					
	High		−0.1 (−2.2;2.0)	8.1 (2.6;13.6)		
# chronic conditions	0 (ref)				<0.001	0.009
	1		−4.6 (−6.2;-3.1)	−1.8 (−5.9;2.2)		
	2		−6.6 (−8.4;-4.7)	−0.5 (−4.8;3.9)		
	3+		−6.8 (−9.9;-3.7)	0.4 (−3.8;4.6)		

^a^The p-value for ‘factor’ relates to testing whether that factor is related to well-being in any way. The p-value for the ‘moderation effect’ relates to testing whether diabetes moderates the relationship between that factor and the well-being score – i.e. whether the relationship is different between individuals with diabetes and those without.

^b^P-values are expected to be small as the only moderation effects that are included in this model are those that had p-values < 0.1 in a model containing all such moderation effects. Model output before exclusion of terms appears in Appendix F of the supplementary material.

There was strong evidence of lower quality of life when having diabetes (−4.2, 95% CI: −9.2,0.9; p-value < 0.001). Similarly, lower scores were associated with not being in a relationship, less formal education, less wealth, lower physical activity and more chronic conditions (see effect sizes in Table 2, all p-values < 0.02). There was some evidence of lower quality of life in males (p = 0.047). The impact of physical activity and number of chronic conditions differed by diabetes status (p-values: 0.028 and 0.009 respectively). High levels of physical activity appear to correspond to higher quality of life only amongst those with diabetes (high versus moderate levels: 8.1, 95% CI: 2.6,13.6. Additional chronic conditions had an effect on quality of life for all participants although the negative effect was more pronounced in those without self-reported diabetes than in those with diabetes.

The results from the zero-inflated negative binomial regression model for WHODAS are shown in Table 3. The odds ratio is the relative change in the odds of a zero disability score (no disability), and the multiplicative effect or exponentiated regression coefficient is the relative change in the average score amongst the remaining individuals. Therefore an odds ratio greater than 1 and multiplicative effect less than 1 together indicate a decrease in disability with a change in the factor (out of the reference category into another category).

**Table 3.** Association of diabetes, socio-demographic characteristics, self-reported health behaviours and co-morbidities with WHODAS (0–36).^c^

		Effect sizes (95% CI)					
			Multiplicative effect/exponentiated regression coefficient			P-values^a^	
Factor	Category	Odds Ratio (OR)	All subjects	Non-diabetic group	Diabetic group	Factor	Moderation effect^b^
Diabetes	No (ref)					<0.001	
	Yes		2.1 (1.5,2.9)				
Sex	Female (ref)					0.125	0.042
	Male			1.0 (0.9,1.2)	0.7 (0.6,1.0)		
Age	50–59 years (ref)					<0.001	
	60–69 years	0.8 (0.5,1.1)	1.1 (1.0,1.2)				
	70+ years	0.5 (0.3,0.9)	1.4 (1.3,1.5)				
Marital status	Single	0.5 (0.3,0.9)		1.1 (0.9,1.3)	1.0 (0.8,1.3)	0.008	0.045
	Married/cohabit (ref)						
	Separated/divorced	0.6 (0.2,1.4)		1.0 (0.9,1.2)	0.6 (0.4,0.9)		
	Widowed	0.6 (0.4,0.8)		1.1 (0.9,1.2)	0.9 (0.7,1.2)		
Years of education	0–5 years (ref)					<0.001	0.000
	6–12 years	1.7 (1.2,2.5)		0.9 (0.8,1.0)	0.9 (0.7,1.1)		
	13+ years	2.6 (1.5,4.5)		0.6 (0.5,0.8)	1.4 (1.0,1.9)		
Same location	Yes (ref)					0.010	
	No		1.1 (1.0,1.3)				
Past work	Yes (ref)					0.005	
	No		1.3 (1.1,1.5)				
Wealth quintile	Lowest		1.2 (1.0,1.4)			0.034	
	Second		1.1 (1.0,1.2)				
	Middle (ref)						
	Fourth		1.0 (0.9,1.1)				
	Highest		0.9 (0.8,1.1)				
Tobacco	No (ref)					0.274	0.108
	Yes			1.0 (0.9,1.2)	0.8 (0.5,1.1)		
Alcohol	No (ref)					0.006	
	Yes		1.2 (1.1,1.4)				
Physical activity	Low			1.4 (1.2,1.6)	0.9 (0.8,1.2)	<0.001	0.006
	Moderate (ref)						
	High			0.8 (0.7,1.0)	0.7 (0.5,1.0)		
# chronic conditions	0 (ref)					<0.001	0.003
	1	0.4 (0.3,0.5)		1.3 (1.2,1.5)	0.9 (0.7,1.2)		
	2	0.2 (0.1,0.3)		1.7 (1.5,1.9)	1.0 (0.8,1.3)		
	3+	0.1 (0.0,0.3)		1.7 (1.4,2.0)	1.0 (0.8,1.4)		

^a^The p-value for ‘factor’ relates to testing whether that factor is related to well-being in any way. The p-value for the ‘moderation effect’ relates to testing whether diabetes moderates the relationship between that factor and the well-being score – i.e. whether the relationship is different between individuals with diabetes and those without.

^b^P-values are expected to be small as the only moderation effects that are included in this model are those that had p-values < 0.1 in a model containing all such moderation effects. Model output before exclusion of terms appears in the Appendix G of the supplementary material.

^c^For the regression model WHODAS score of 0–36 was retained.

Diabetes was associated with greater disability (multiplicative effect: 2.1; 95% CI: 1.59,2.9; p-value: < 0.001). There was also greater disability associated with being older, being single or widowed (although there are mixed results for those with diabetes), having more chronic conditions and having lower education levels (all p-values < 0.01, see Table 3 for effect sizes). Greater disability is also associated with no past employment, and having less wealth, any alcohol use and lower physical activity levels (multiplicative effects up to 1.3; p-values < 0.05). The impact of marital status, education, physical activity and the number of chronic conditions differed by diabetes status (p-values < 0.05). Also, an increase in the number of chronic conditions is associated with large reductions in the odds of zero disability (OR for 3+ conditions versus 0 conditions: 0.1; 95% CI: 0.0, 0.3), thus implying greater disability. There was a clear increase in the disability of the remaining individuals amongst those without diabetes (multiplicative effect: 1.7; 95% CI: 1.4,2.0) but not in those with diabetes.

## Discussion

In this analysis of SAGE South Africa Wave 1 data, a 9.2% prevalence rate of self-reported diabetes was found. Individuals with diabetes had at least twofold higher rates of coexisting chronic conditions than those without diabetes. In addition, diabetes status per se was associated with poor quality of life and disability, as were socio-economic status (low education), being in a low wealth quintile, having a poor employment history, marital status (not being in a partnership), lifestyle habits (low physical activity, history of alcohol use) and co-morbid conditions. In accordance with previous studies, we found that having  diabetes [20–22], having lower formal education levels [23–25], being in a low socioeconomic group [26,27], not being in a marital relationship [28,29] and not having worked [30,31] were significantly associated with poor quality of life and a high level of disability, but unlike findings in other studies [32–35], being older and being female were not associated with a high level of disability.20–22 23–25 26,27 28,29]30,31 32–35 The differences in findings in this study compared to others may relate to the use of different instruments to assess the quality of life and disability, as well as the use of diabetes-specific and generic measures across studies. In short, the study findings support a relationship between self-reported diabetes, QoL and disability, while controlling for a variety of factors such as socio-demographic characteristics, health risk behaviours and coexisting chronic conditions.

The prevalence rate of self-reported diabetes in the study reflects some factors, including the participants’ low level of knowledge on diabetes, and access to diagnostic and care facilities. South African epidemiological studies have established about a 60% prevalence rate for previously diagnosed diabetes in urban areas, compared to a rate of about 15% in rural areas. The observation in our study that diabetes was more common in higher than in lower wealth quintiles supports previous findings in urban black Africans in Cape Town [36]. However, our observation may similarly indicate this group’s greater access to health care as well as a change in lifestyle habits, such as poor, high-sugar diets and low physical activity, often accompanying urbanisation.

Studies have shown that co-morbid conditions, more than other factors, determined the quality of life of people with diabetes. For example, Rubin et al. noted that the presence of co-morbid conditions could further interact with the severity of the disease and its complications to strongly influence different domains of quality of life [37]. We did not deconstruct the composite WHOQoL score to examine the contributions of the different domains, but the overall score suggested that the impact of co-morbidities on QoL was considerable in older respondents regardless of diabetes status. The magnitude of the effect size of QoL reductions differed statistically between 4.6 and 6.8 points as the number of chronic conditions increased from 1 to 3 or more. A gradient in QoL pertaining to the number of chronic diseases in the non-diabetes group was not found in individuals with diabetes which is probably due to (a) the relatively small size sample of the diabetes group (n = 341), and (b) use of a self-report questionnaire to determine the status of the chronic condition and possible underestimation of prevalence. Nevertheless, co-morbidities can profoundly affect older patients’ ability to care for their conditions and can pose significant barriers to lifestyle changes and accessing needed care [38].

The results of the study showed a statistically significant impact of the level of education on the quality of life of older people with diabetes. Other studies have similarly shown a positive correlation between levels of education and quality of life. It is conceivable that those with a higher education will have a better understanding of the illness and its effect on them, will be in a better financial position to avail themselves of quality treatment and will be more likely to adhere to treatment regimens and self-management of the condition [39].

Study participants with and without diabetes who had a high level of physical activity were found to have higher QoL scores. We are unable to determine whether this finding is due to cause or effect; it is well recognised that exercise nonetheless is associated with higher subjective well-being [40,41]. Being sedentary was found to impact the quality of life, which is consistent with other studies: for example, in older individuals, even light-intensity physical activity is related to higher self-rated physical health and psychosocial well-being [42]. Notably, the prevalence of a low level of physical activity in the South Africa data was higher than in any other SAGE country (China, Ghana, India, Mexico, Russian Federation) [43]. Although age and diabetes conspire to decrease fitness and strength, physical activity interventions improve functional status in older adults with and without diabetes [44].

In previous studies, diabetes represented not only a risk factor for disability, but was associated with a wide range of impairments and co-morbidities predisposing to loss of autonomy [45]. This association is partly explained in our study by the impact of diabetes itself, socio-demographic characteristics, health risk factors, health behaviours and co-morbidity on disability. Reasons for this effect are not clear. Findings from an earlier study showed a 100–150% increased incidence of disability among older women with diabetes compared to non-diabetic age peers [46]. Indeed, women with diabetes had a 78% increased risk of mobility-related disability and a 65% higher risk of activities of daily living (ADL) disability [47]. In a sample of over 1000 managed-care patients with diabetes with lower formal education and low physical activity, each was associated with disability. However, the pathway between diabetes and physical disability is multifactorial, and it is not possible to differentiate cause and effect in a cross-sectional study [47]. This study investigated the relation of the overall disability score and not to domain-specific disability as they relate to diabetes and other health and socio-economic characteristics.

The present study highlights a need to fill gaps in knowledge towards improving quality of life and meeting care needs in older adults with diabetes. A dearth of research on leading health issues such as diabetes in older adults in sub-Saharan Africa countries impacts health policy and planning. Evidence-based policy and planning on services for older clients is underdeveloped or lacking in the subcontinent, partially due to epidemiological and demographic transitions being recent and more rapid than in high-income countries [48]. Qualitative research is required to address research questions in depth relating to the impact of factors such as retirement, low income, living alone, age-friendly vs. ageist attitudes of health professionals towards older clients, and the promotion of physical activity. Comparison of study outcomes on quality of life is a challenge due to the differing content validity of instruments used in different studies.

This study has both strengths and limitations. The strength of the study is that it was able to draw on data from SAGE which has a representative sample of South Africa. Quality of life is influenced by a person’s experience, beliefs, expectations and perceptions which are in turn influenced by socio-economic status, cultural identity and literacy level [49,50]. It is for this reason that a standardised and well-tested instrument was used. Ethnicity may be used as a proxy for poor socio-economic circumstances; in this study, we have a number of direct measures of socio-economic status, such as wealth quintile and education. Due to the high number of missing values (13%) this study opted not to use race which is the proxy in other similar studies. Additional limitations are that SAGE was not specific for the study of diabetes. Glycated haemoglobin data from dried blood spots collected as part of SAGE and linked to the survey data would help identify those individuals with undiagnosed diabetes and strengthen future analyses. Specific complications associated with diabetes which cause disability and impact quality of life were not recorded in SAGE. As the data are cross-sectional, causality cannot be attributed from the recorded associations between diabetes and quality of life or disability. More importantly, longitudinal data in South Africa to examine the relationship more fully are required, and further qualitative and quantitative research methods should be used to assess and refine existing instruments, and to articulate and describe measure outcomes.

## Conclusion

Diabetes negatively impacts quality of life and disability status in older South African adults. As the number of older adults with diabetes in African countries is anticipated to increase significantly in the coming decades, sustainable policies are needed to promote healthy ageing at local and national levels. Further appropriate health care for older adults with diabetes can prevent or reduce disability, and avoid diminished quality of life associated with poor diabetes management which results in disabling complications such as stroke, kidney disease, amputations and impaired vision.

## Paper context

Quality of life and disability are powerful predictors of a person’s capacity to manage and maintain longer-term health and well-being. This study aimed to fill a gap in knowledge regarding the impact of diabetes on the QoL and disability of South Africans aged 50 years and older. We recommended sustainable policies to promote healthy eating behaviours, increase physical activity and manage health conditions, across the life course.
