# Supplementary material for: Diabetes in South African older adults: prevalence and impact on quality of life and functional disability – as assessed using SAGE Wave 1 data
Source: Glob Health Action. 2018 Apr 27;11(1):1449924. doi: 10.1080/16549716.2018.1449924 (PMC5933282; doi:10.1080/16549716.2018.1449924)
Supplement: Supplementary material [file ZGHA_A_1449924_SM4578.docx]

**Supplementary Materials**

**Appendix A: Summary statistics for WHOQoL**

**Table A1. Summary statistics (mean and standard deviation) for WHOQOL (0-100) by covariates and diabetes status**

|  |  | **WHOQoL mean (std deviation)** | | |
| --- | --- | --- | --- | --- |
| **Factor** | **Category** | **All subjects** | **Diabetics** | **Non-diabetics** |
| **All subjects** |  | 47.4 (12.8) | 46.9 (11.3) | 47.4 (13.0) |
| **Diabetes** | **No** | 47.4 (13.0) |  | 47.4 (13.0) |
|  | **Yes** | 46.9 (11.3) | 46.9 (11.3) |  |
| **Sex** | **Female** | 46.4 (13.0) | 46.5 (11.3) | 46.4 (13.2) |
|  | **Male** | 48.6 (12.5) | 47.9 (11.3) | 48.7 (12.6) |
| **Age** | **50-59 years** | 47.7 (12.8) | 46.5 (11.6) | 47.8 (12.9) |
|  | **60-69 years** | 47.2 (12.8) | 45.7 (10.9) | 47.4 (13.0) |
|  | **70+ years** | 46.7 (12.9) | 49.2 (11.3) | 46.3 (13.1) |
| **Marital status** | **Single** | 44.1 (12.9) | 42.7 (11.1) | 44.3 (13.1) |
|  | **Married/cohabiting** | 49.5 (12.3) | 49.2 (10.9) | 49.5 (12.5) |
|  | **Separated/divorced** | 44.2 (14.0) | 44.9 (9.6) | 44.2 (14.2) |
|  | **Widowed** | 45.2 (12.7) | 45.7 (11.4) | 45.1 (12.9) |
| **Years of education** | **0-5 years** | 44.0 (11.6) | 42.6 (9.5) | 44.1 (11.8) |
|  | **6-12 years** | 48.4 (12.6) | 48.3 (11.4) | 48.4 (12.8) |
|  | **13+ years** | 55.7 (11.3) | 56.0 (7.7) | 55.6 (11.5) |
| **Ethnicity** | **African/black** | 44.9 (11.9) | 44.5 (11.2) | 44.9 (11.9) |
|  | **White** | 58.6 (10.5) | 55.7 (6.7) | 59.0 (10.9) |
|  | **Coloured** | 50.8 (12.2) | 50.8 (11.2) | 50.8 (12.3) |
|  | **Indian/Asian** | 49.5 (12.4) | 48.7 (10.0) | 49.7 (13.1) |
| **Same location** | **Yes** | 46.8 (12.0) | 47.7 (10.7) | 46.8 (12.2) |
|  | **No** | 47.8 (13.5) | 45.9 (12.5) | 47.9 (13.6) |
| **Past work** | **Yes** | 48.2 (12.5) | 48.0 (11.4) | 48.2 (12.7) |
|  | **No** | 42.3 (13.3) | 41.6 (9.1) | 42.4 (13.7) |
| **Wealth quintile** | **poorest** | 40.2 (11.7) | 37.9 (9.4) | 40.3 (11.8) |
|  | **second** | 44.1 (11.7) | 41.0 (12.3) | 44.4 (11.6) |
|  | **middle** | 46.8 (12.3) | 48.7 (10.4) | 46.7 (12.4) |
|  | **fourth** | 49.5 (11.5) | 46.7 (10.5) | 49.8 (11.5) |
|  | **richest** | 55.5 (11.5) | 52.6 (8.9) | 55.9 (11.8) |
| **Tobacco** | **No** | 47.4 (12.7) | 47.0 (11.4) | 47.5 (12.9) |
|  | **Yes** | 47.2 (13.1) | 46.9 (10.9) | 47.2 (13.2) |
| **Alcohol** | **No** | 47.7 (12.5) | 47.7 (11.2) | 47.7 (12.6) |
|  | **Yes** | 46.3 (13.8) | 41.9 (10.8) | 46.6 (13.9) |
| **Physical activity** | **Low** | 46.0 (12.8) | 44.8 (11.2) | 46.1 (13.0) |
|  | **Moderate** | 49.8 (12.5) | 47.3 (8.9) | 50.0 (12.8) |
|  | **High** | 49.8 (12.3) | 55.0 (10.6) | 49.4 (12.3) |
| **Arthritis** | **No** | 48.5 (12.6) | 47.3 (11.2) | 48.5 (12.7) |
|  | **Yes** | 44.0 (12.8) | 46.5 (11.4) | 43.5 (13.0) |
| **Stroke** | **No** | 47.6 (12.7) | 47.3 (11.4) | 47.6 (12.9) |
|  | **Yes** | 41.9 (13.8) | 42.2 (8.9) | 41.8 (14.6) |
| **Angina** | **No** | 47.5 (12.8) | 47.1 (11.3) | 47.6 (12.9) |
|  | **Yes** | 44.2 (13.2) | 45.9 (11.3) | 43.7 (13.7) |
| **Lung disease** | **No** | 47.5 (12.7) | 47.5 (11.2) | 47.5 (12.9) |
|  | **Yes** | 42.9 (15.2) | 41.0 (10.4) | 43.6 (16.6) |
| **Asthma** | **No** | 47.7 (12.7) | 47.6 (11.3) | 47.7 (12.9) |
|  | **Yes** | 41.5 (13.1) | 40.5 (9.2) | 41.7 (13.8) |
| **Depression** | **No** | 47.5 (12.8) | 47.2 (11.4) | 47.6 (12.9) |
|  | **Yes** | 41.4 (12.6) | 42.2 (8.7) | 41.3 (13.3) |
| **Hypertension** | **No** | 47.7 (13.0) | 47.3 (10.6) | 47.8 (13.1) |
|  | **Yes** | 46.5 (12.5) | 46.8 (11.6) | 46.4 (12.7) |
| **Cataracts** | **No** | 47.3 (12.9) | 46.6 (11.2) | 47.4 (13.0) |
|  | **Yes** | 49.1 (11.5) | 51.3 (9.9) | 48.2 (12.0) |
| **# chronic conditions** | **0** | 49.4 (12.4) | 47.4 (9.4) | 49.5 (12.5) |
|  | **1** | 45.6 (12.9) | 47.0 (12.6) | 45.4 (13.0) |
|  | **2** | 45.6 (13.2) | 47.6 (11.3) | 45.0 (13.6) |
|  | **3+** | 44.0 (12.5) | 47.1 (9.3) | 42.9 (13.3) |

**Appendix B: Summary statistics for WHODAS**

**Table A2. Summary statistics (mean and standard deviation) for WHODAS (0-36) by covariates and diabetes status**

|  |  | **WHODASi-r mean (std deviation)** | | |
| --- | --- | --- | --- | --- |
| **Factor** | **Category** | **All subjects** | **Diabetics** | **Non-diabetics** |
| **All subjects** |  | 20.5 (20.3) | 25.6 (19.1) | 20.2 (20.3) |
| **Diabetes** | **No** | 20.2 (20.3) |  | 20.2 (20.3) |
|  | **Yes** | 25.6 (19.1) | 25.6 (19.1) |  |
| **Sex** | **Female** | 22.7 (20.6) | 27.2 (18.7) | 22.2 (20.7) |
|  | **Male** | 17.8 (19.6) | 22.4 (19.5) | 17.6 (19.6) |
| **Age** | **50-59 years** | 15.8 (16.9) | 19.4 (15.8) | 15.8 (17.0) |
|  | **60-69 years** | 22.0 (21.2) | 29.2 (20.6) | 21.3 (21.1) |
|  | **70+ years** | 30.3 (22.8) | 29.7 (19.1) | 30.3 (23.1) |
| **Marital status** | **Single** | 21.1 (18.9) | 27.1 (15.7) | 20.8 (19.2) |
|  | **Married/cohabiting** | 17.8 (19.1) | 23.1 (18.4) | 17.4 (19.2) |
|  | **Separated/divorced** | 22.6 (22.8) | 12.8 (10.0) | 23.4 (23.3) |
|  | **Widowed** | 26.2 (21.8) | 29.2 (20.8) | 26.0 (21.7) |
| **Years of education** | **0-5 years** | 24.3 (21.4) | 30.0 (20.9) | 24.1 (21.4) |
|  | **6-12 years** | 18.7 (19.0) | 22.9 (17.8) | 18.3 (19.1) |
|  | **13+ years** | 11.6 (14.2) | 27.8 (15.3) | 10.5 (13.5) |
| **Ethnicity** | **African/black** | 21.8 (20.4) | 26.5 (18.5) | 21.6 (20.6) |
|  | **White** | 12.6 (15.9) | 27.6 (16.3) | 10.6 (14.8) |
|  | **Coloured** | 18.9 (19.7) | 13.9 (18.5) | 19.5 (19.7) |
|  | **Indian/Asian** | 26.7 (22.2) | 32.6 (20.9) | 25.2 (22.1) |
| **Same location** | **Yes** | 20.1 (19.8) | 23.5 (17.8) | 20.0 (19.9) |
|  | **No** | 21.8 (20.9) | 29.2 (21.2) | 21.1 (20.7) |
| **Past work** | **Yes** | 19.4 (19.4) | 23.0 (17.3) | 19.0 (19.5) |
|  | **No** | 27.5 (23.8) | 39.1 (22.0) | 26.9 (23.7) |
| **Wealth quintile** | **poorest** | 23.9 (21.5) | 35.2 (22.1) | 23.8 (21.4) |
|  | **second** | 21.6 (21.5) | 29.5 (16.3) | 21.0 (21.6) |
|  | **middle** | 21.4 (20.1) | 22.3 (18.5) | 21.3 (20.3) |
|  | **fourth** | 20.0 (18.8) | 24.1 (19.2) | 19.5 (18.7) |
|  | **richest** | 16.2 (18.7) | 23.2 (18.3) | 15.3 (18.6) |
| **Tobacco** | **No** | 20.9 (20.4) | 27.5 (18.1) | 20.1 (20.5) |
|  | **Yes** | 20.2 (20.1) | 18.4 (21.0) | 20.3 (20.0) |
| **Alcohol** | **No** | 20.7 (20.4) | 25.2 (18.9) | 20.2 (20.5) |
|  | **Yes** | 20.6 (20.1) | 28.3 (20.1) | 20.2 (20.0) |
| **Physical activity** | **Low** | 24.7 (22.2) | 26.2 (20.1) | 24.5 (22.4) |
|  | **Moderate** | 16.6 (16.7) | 27.5 (13.7) | 15.5 (16.6) |
|  | **High** | 13.4 (14.3) | 17.5 (14.0) | 13.1 (14.3) |
| **Arthritis** | **No** | 17.4 (18.9) | 21.4 (17.7) | 17.2 (19.0) |
|  | **Yes** | 30.5 (21.1) | 30.7 (19.4) | 30.4 (21.4) |
| **Stroke** | **No** | 20.0 (19.8) | 24.5 (18.4) | 19.5 (19.9) |
|  | **Yes** | 37.5 (23.6) | 39.6 (22.8) | 37.0 (23.8) |
| **Angina** | **No** | 20.2 (20.1) | 25.0 (18.5) | 19.8 (20.2) |
|  | **Yes** | 28.9 (21.1) | 30.1 (22.3) | 28.5 (20.8) |
| **Lung disease** | **No** | 20.5 (20.2) | 24.6 (18.8) | 20.1 (20.3) |
|  | **Yes** | 27.9 (20.7) | 36.5 (18.7) | 24.8 (20.7) |
| **Asthma** | **No** | 20.2 (20.2) | 25.5 (19.0) | 19.7 (20.3) |
|  | **Yes** | 29.4 (19.5) | 26.4 (20.3) | 30.1 (19.4) |
| **Depression** | **No** | 20.4 (20.3) | 24.8 (18.9) | 20.0 (20.4) |
|  | **Yes** | 29.9 (18.0) | 39.6 (17.2) | 27.8 (17.6) |
| **Hypertension** | **No** | 18.3 (19.6) | 21.4 (18.7) | 18.2 (19.6) |
|  | **Yes** | 26.0 (20.9) | 27.4 (19.0) | 25.6 (21.3) |
| **Cataracts** | **No** | 20.4 (20.3) | 25.3 (18.9) | 19.9 (20.4) |
|  | **Yes** | 25.4 (18.5) | 23.2 (18.3) | 26.2 (18.6) |
| **# chronic conditions** | **0** | 14.9 (18.0) | 20.8 (16.1) | 14.7 (18.0) |
|  | **1** | 23.9 (20.2) | 23.5 (19.0) | 23.9 (20.3) |
|  | **2** | 28.6 (21.3) | 25.3 (19.2) | 29.6 (21.8) |
|  | **3+** | 33.5 (20.3) | 30.8 (18.7) | 34.4 (20.8) |

**Appendix C: Distribution of WHOQoL (0-100 scale)**

**Figure C1. Histogram of WHOQoL scores, with superimposed fitted normal distribution**

**Figure C2. Box-and whisker plots of WHOQoL scores (0-100 scale), stratified by diabetes status, sociodemographic factors, health risk behaviours, and number of chronic conditions**

Same location=usual place of residence; Wealth quantile: poorest=lowest, richest=highest

**Appendix D: Distribution of WHODAS (0-36 scale)**

**Figure D1. Histogram of WHODAS scores, with superimposed fitted negative binomial distribution and zero-inflated negative binomial distribution**

**Figure D2. Box-and whisker plots of WHODAS scores (0-36 scale), stratified by diabetes status, sociodemographic factors, health risk behaviours, and number of chronic conditions**

Same location=usual place of residence; Wealth quantile: poorest=lowest, richest=highest

**Appendix E: Methodology**

Data were analysed using R (version 3.1.3) and Stata (Stata/MP 13.1). The sampling design and probability weights were taken into account using R’s ‘survey’ package and Stata’s suite of ‘svy’ commands .

We assessed the association of diabetes and other factors with WHOQOL and WHODAS, as well as the moderation effects of diabetes, using generalised linear models. WHOQoL and WHODAS were considered in turn.

For WHOQOL, a standard multiple linear regression model was used. Estimated effect sizes captured the absolute change in the mean WHOQOL score as a factor changed from the reference category to the indicated category, holding all else equal – termed additive effects in the text. These impacts were allowed to differ by diabetes status. Initially diabetes was allowed to moderate all effects (see Appendix F for the full model), but in the main text a trimmed model is presented, where the moderation effects included are those where the initial model suggested evidence of moderation (p-values <0.1).

For WHODAS, a zero-inflated negative binomial regression model was used to describe disability on a 0-36 count scale, with higher values indicating greater disability level. Within this framework, disability scores are split into two distinct groups. Firstly, there are the excess of 0 scores, corresponding to no disability, that result in a peak of scores at 0. This clustering of 0 values needs to be explicitly accounted for in the statistical model. Secondly, there are the remaining scores which range from 0 upwards and follow a standard statistical distribution (a negative-binomial distribution in this case). Two sets of effect sizes are thus reported: Odds ratios (ORs) for the excess 0 scores, and multiplicative effects, a term used here to indicate the ratio change in the mean of the remaining scores. In the initial model, each factor was allowed to (independently) impact the OR, and the multiplicative effect allowed to differ by diabetes status (see Appendix G for the full model). In the main text, a trimmed model is presented: the moderation effects included are those where the initial model suggested evidence of moderation (p-values <0.1); an OR was included for a factor only where the initial model suggested evidence for this (p-values <0.1). Changes in factors that are associated with a decrease in disability can be identified by an OR greater than 1 (i.e. more scores are clustered at 0) and a multiplicative effect less than 1 (i.e. after accounting for and removing the excess of 0 scores, the average of the remaining scores is also smaller). Conversely, when the OR is less than 1 and multiplicative effect greater than 1, there are fewer excess 0 scores and the remaining scores are centered around a larger value, and there is greater disability.

Model fit was assessed using residual plots (not shown here) and comparisons of observed and model-fitted means (see Appendix H for some outputs).

Confidence intervals (CIs) for estimated model parameters are based on Wald-type intervals. The significance of terms was assessed using Wald tests, where small p-values suggest evidence of relationships (or rather evidence against the hypotheses of no relationships).

List-wise deletion of observations with missing values resulted in 2848 observations for WHOQOL and 2866 observations for WHODAS. The most common sources of missing values were: 570 for education, 492 for place of residence and 123 for physical activity.

Estimated effect sizes and CIs are reported in the results, as well as two key p-values for each factor: (1) The p-value for ‘factor’ relates to testing whether that factor is related to well-being (WHOQOL or WHODAS) in any way. (2) The p-value for the ‘moderation effect’ relates to testing whether diabetes moderates the relationship between that factor and the well-being score – i.e. whether the relationship is different between individuals with diabetes and those without.

Because testing for moderation is of interest, the dataset was not stratified into individuals with diabetes and those without, but rather all data used in the model.

**Appendix F: Untrimmed regression model for WHOQoL**

**Table F1: Association of diabetes, sociodemographic characteristics, self-reported health behaviours and comorbidities with WHOQOL (0-100) – original model before removing terms**

|  |  | **Additive effect / regression coefficient (95% CI)** | | **P-value^1^** | |
| --- | --- | --- | --- | --- | --- |
| **Factor** | **Category** | **Non-diabetic group** | **Diabetic**  **group** | **Factor** | **Moderation effect** |
| **Diabetes** | **No (ref)** |  | 4.6 (-1.4;10.7) | <0.001 | N/A |
|  | Yes | -4.6 (-10.7;1.4) |  |  |  |
| **Sex** | **Female (ref)** |  |  | 0.086 | 0.373 |
|  | Male | -1.6 (-3.0;-0.2) | -0.2 (-3.0;2.7) |  |  |
| **Age** | **50-59 years (ref)** |  |  | 0.177 | 0.975 |
|  | 60-69 years | 1.7 (0.3;3.2) | 1.3 (-2.4;5.0) |  |  |
|  | 70+ years | 1.4 (-0.4;3.3) | 1.1 (-2.9;5.1) |  |  |
| **Marital status** | Single | -2.6 (-4.9;-0.3) | -2.4 (-6.2;1.4) | 0.052 | 0.978 |
|  | **Married/cohabiting (ref)** |  |  |  |  |
|  | Separated/divorced | -1.2 (-4.3;1.9) | -1.1 (-6.8;4.5) |  |  |
|  | Widowed | -2.6 (-4.3;-0.9) | -1.7 (-5.2;1.7) |  |  |
| **Years of education** | **0-5 years (ref)** |  |  | 0.001 | 0.102 |
|  | 6-12 years | 2.1 (0.4;3.8) | 2.7 (-0.7;6.1) |  |  |
|  | 13+ years | 4.4 (1.2;7.6) | 12.5 (5.4;19.7) |  |  |
| **Same location** | **Yes (ref)** |  |  | 0.704 | 0.411 |
|  | No | 0.4 (-1.2;2.0) | -1.1 (-4.3;2.1) |  |  |
| **Past work** | **Yes (ref)** |  |  | 0.003 | 0.086 |
|  | No | -4.3 (-6.7;-1.9) | -0.5 (-4.3;3.3) |  |  |
| **Wealth quintile** | lowest | -6.8 (-9.2;-4.5) | -9.3 (-14.3;-4.3) | <0.001 | 0.075 |
|  | second | -2.8 (-5.2;-0.3) | -5.8 (-12.0;0.5) |  |  |
|  | **middle (ref)** |  |  |  |  |
|  | fourth | 0.6 (-1.5;2.6) | -3.4 (-8.0;1.2) |  |  |
|  | highest | 7.0 (4.3;9.7) | 0.4 (-3.4;4.2) |  |  |
| **Tobacco** | **No (ref)** |  |  | 0.228 | 0.138 |
|  | Yes | 0.2 (-1.6;2.0) | 3.4 (-0.5;7.3) |  |  |
| **Alcohol** | **No (ref)** |  |  | 0.14 | 0.924 |
|  | Yes | -1.6 (-3.6;0.3) | -1.8 (-4.8;1.2) |  |  |
| **Physical activity** | Low | -3.3 (-5.3;-1.3) | -1.2 (-4.6;2.3) | <0.001 | 0.023 |
|  | **Moderate (ref)** |  |  |  |  |
|  | High | -0.1 (-2.2;2.0) | 8.0 (2.7;13.4) |  |  |
| **# chronic conditions** | **0 (ref)** |  |  | <0.001 | 0.001 |
|  | 1 | -4.7 (-6.2;-3.1) | -2.2 (-5.7;1.4) |  |  |
|  | 2 | -6.6 (-8.5;-4.8) | -0.4 (-4.4;3.6) |  |  |
|  | 3+ | -6.9 (-10.0;-3.8) | 1.6 (-2.3;5.5) |  |  |

^1^ The p-value for ‘factor’ relates to testing whether that factor is related to well-being in any way. The p-value for the ‘moderation effect’ relates to testing whether diabetes moderates the relationship between that factor and the well-being score – i.e. whether the relationship is different between individuals with diabetes and those without.

Same location=usual place of residence

**Appendix G: Untrimmed regression model for WHODAS**

**Table G1: Association of diabetes, sociodemographic characteristics, self-reported health behaviours and comorbidities with WHODAS (0-36) – original model before removing terms**

|  |  | **Effect sizes (95% CI)** | | | **P-value^1^** | |
| --- | --- | --- | --- | --- | --- | --- |
| **Factor** | **Category** | **Odds Ratio**  **(OR)** | **Multiplicative effect / exponentiated regression coefficient** | | **Factor** | **Moderation effect** |
|  |  |  | **Non-diabetic group** | **Diabetic**  **group** |  |  |
| **Diabetes** | **No (ref)** | 1.9 (0.6,5.5) |  | 0.6 (0.4,0.8) | <0.001 | N/A |
|  | Yes | 0.5 (0.2,1.6) | 1.7 (1.2,2.5) |  |  |  |
| **Sex** | **Female (ref)** |  |  |  | 0.119 | 0.031 |
|  | Male | 1.1 (0.8,1.6) | 1.0 (0.9,1.2) | 0.7 (0.5,1.0) |  |  |
| **Age** | **50-59 years (ref)** |  |  |  | <0.001 | 0.846 |
|  | 60-69 years | 0.8 (0.5,1.1) | 1.1 (0.9,1.2) | 1.1 (0.8,1.5) |  |  |
|  | 70+ years | 0.6 (0.3,0.9) | 1.4 (1.2,1.5) | 1.5 (1.1,2.0) |  |  |
| **Marital status** | Single | 0.5 (0.3,0.9) | 1.1 (0.9,1.3) | 1.0 (0.8,1.4) | 0.015 | 0.047 |
|  | **Married/cohabiting (ref)** |  |  |  |  |  |
|  | Separated/divorced | 0.5 (0.2,1.2) | 1.0 (0.9,1.2) | 0.6 (0.3,0.9) |  |  |
|  | Widowed | 0.5 (0.3,0.9) | 1.1 (0.9,1.2) | 0.9 (0.7,1.2) |  |  |
| **Years of education** | **0-5 years (ref)** |  |  |  | <0.001 | 0.000 |
|  | 6-12 years | 2.0 (1.4,2.9) | 0.9 (0.8,1.0) | 0.9 (0.7,1.1) |  |  |
|  | 13+ years | 3.2 (1.8,5.9) | 0.6 (0.5,0.8) | 1.5 (1.0,2.3) |  |  |
| **Same location** | **Yes (ref)** |  |  |  | 0.005 | 0.121 |
|  | No | 1.3 (0.9,1.8) | 1.1 (1.0,1.3) | 1.4 (1.1,1.8) |  |  |
| **Past work** | **Yes (ref)** |  |  |  | 0.004 | 0.183 |
|  | No | 1.0 (0.5,1.9) | 1.3 (1.0,1.5) | 1.5 (1.2,2.1) |  |  |
| **Wealth quintile** | poorest | 1.3 (0.7,2.2) | 1.2 (1.0,1.4) | 1.3 (0.9,2.0) | 0.087 | 0.870 |
|  | second | 1.1 (0.7,1.9) | 1.1 (1.0,1.2) | 1.3 (0.9,1.9) |  |  |
|  | **middle (ref)** |  |  |  |  |  |
|  | fourth | 0.6 (0.3,1.0) | 1.0 (0.8,1.1) | 1.0 (0.7,1.4) |  |  |
|  | richest | 0.9 (0.5,1.5) | 0.9 (0.8,1.1) | 1.0 (0.7,1.4) |  |  |
| **Tobacco** | **No (ref)** |  |  |  | 0.361 | 0.077 |
|  | Yes | 1.0 (0.7,1.5) | 1.0 (0.9,1.2) | 0.7 (0.5,1.0) |  |  |
| **Alcohol** | **No (ref)** |  |  |  | 0.033 | 0.537 |
|  | Yes | 1.0 (0.6,1.5) | 1.2 (1.0,1.4) | 1.4 (0.9,2.0) |  |  |
| **Physical activity** | Low | 1.0 (0.6,1.7) | 1.4 (1.2,1.6) | 0.9 (0.7,1.1) | <0.001 | 0.002 |
|  | **Moderate (ref)** |  |  |  |  |  |
|  | High | 0.7 (0.4,1.3) | 0.8 (0.7,1.0) | 0.7 (0.5,1.0) |  |  |
| **# chronic conditions** | **0 (ref)** |  |  |  | <0.001 | 0.003 |
|  | 1 | 0.4 (0.2,0.5) | 1.3 (1.2,1.5) | 0.9 (0.6,1.1) |  |  |
|  | 2 | 0.2 (0.1,0.3) | 1.7 (1.5,1.9) | 1.0 (0.8,1.4) |  |  |
|  | 3+ | 0.1 (0.0,0.3) | 1.7 (1.4,2.0) | 1.0 (0.7,1.4) |  |  |

^1^ The p-value for ‘factor’ relates to testing whether that factor is related to well-being in any way. The p-value for the ‘moderation effect’ relates to testing whether diabetes moderates the relationship between that factor and the well-being score – i.e. whether the relationship is different between individuals with diabetes and those without.

Same location=usual place of residence

**Appendix H: Observed versus model fitted average scores**

**Table H1: Observed versus model-fitted (trimmed model) mean WHOQoL (0-100 scale) scores**


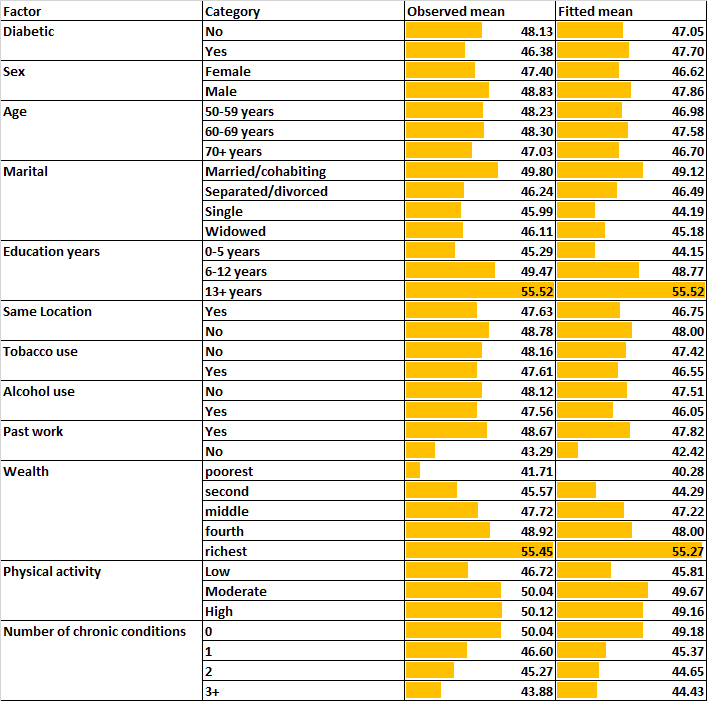


Same location=usual place of residence; Wealth quantile: poorest=lowest, richest=highest

**Table H2: Observed versus model-fitted (trimmed model) mean WHODAS (0-36 scale) scores**


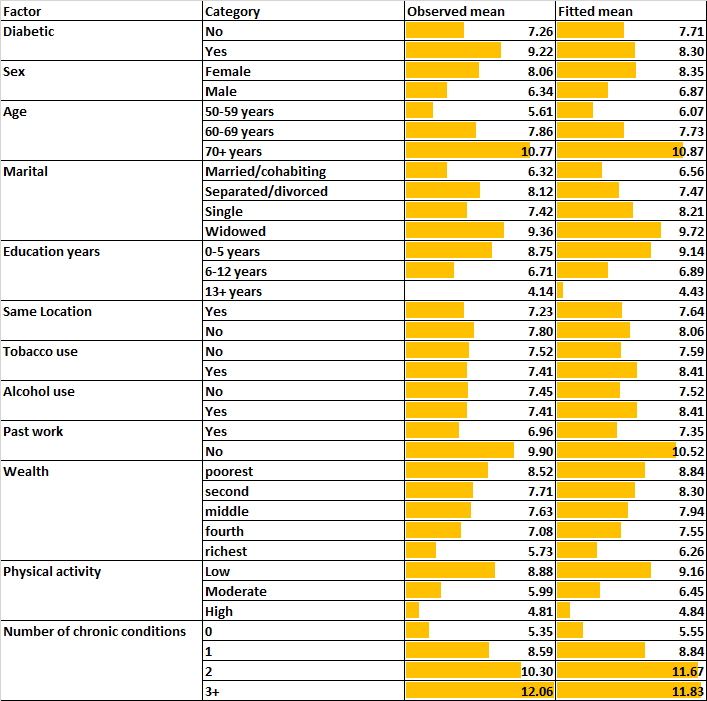


Same location=usual place of residence; Wealth quantile: poorest=lowest, richest=highest
